# Views of students on qualities expected of their lecturers: a case study of the University of Medical Sciences, Ondo city, Nigeria

**DOI:** 10.11604/pamj.2020.35.64.16597

**Published:** 2020-03-03

**Authors:** Wilfred Aghahekokhian Iguodala, Friday Ebodaghe Okonofua, Oluseyi Ademola Adejumo, Oluyemi Adewole Okunlola

**Affiliations:** 1 Academic Planning, the University of Medical Sciences (UNIMED), Ondo city, Ondo State, Nigeria; 2 The University of Medical Sciences (UNIMED), Ondo city, Ondo State, Nigeria; 3 Department of Medicine, the University of Medical Sciences (UNIMED), Ondo city, Ondo State, Nigeria

**Keywords:** University of Medical Sciences, students, teachers, traits, Ondo State, Nigeria

## Abstract

Although the assessment of teachers by students has been introduced into tertiary educational development in Nigeria, very limited information exists on students’ expectations of their teachers. We investigated this component among a cohort of newly admitted students at the University of Medical Sciences in Ondo State, South-West Nigeria. This was a descriptive quantitative study consisting of a community-interactive session with students at the 100 and 200 levels of the University. Three hundred (300) students participated in the session. We first explained the purpose of the study. Thereafter, the students individually completed a semi-structured questionnaire that elicited information on their views on the qualities they expected of their lecturers. The results were analyzed quantitatively with SPSS version 21. Of the 300 students, 204 (64.0%) completed the questionnaire. Friendliness and congeniality (46.1%), good classroom management (38.7%), good sense of humor (36.3%), good communication skills (33.3%) and expertise (32.8%) were the five most desirable qualities of good lecturers listed by the students. By contrast, the five qualities which rated lowest in the assessment were equity (4.4%), mentoring capacity (4.9%), enthusiasm (6.9%), encouraging students to succeed (7.8%) and approachability (8.3%). We conclude that students at the University of Medical Sciences look out for personal social relationships with their teachers during curricular delivery. We recommend that approaches to address these concerns should be incorporated into the design of training programs for teachers and in protocols for students’ evaluation of teachers in this university and others in similar circumstances.

## Introduction

The assessment of teachers by students has repeatedly featured as an indicator for measuring the quality of curricular delivery in tertiary institutions [1–4]. While this method has gained ascendancy in many parts of the developed world, not much emphasis has been placed on understanding what students expect of their teachers in terms of curricular delivery and social interactions in Nigeria. Tertiary education in Nigeria as in other countries around the world has over the years, taken a new dimension. The modern trend is centered on developing the pedagogical skills of teaching as opposed to anagogical teaching methods. In particular, the modern trend is to shift from the traditional didactic, teacher-focused teaching to student-centered methodologies that encourage the active engagement of students in the learning process [5]. More recently, new multimedia technologies have been used in teaching, and new methods and procedures of assessment have been developed and utilized. This is consistent with the constructivist learning theories, which considers the learner as an active partner in the process of learning, teaching and assessment [6]. Teaching especially in tertiary institutions is now considered as involving not just the transmission of content and skills but as a major preparatory phase for students who are to be launched into the society as major impact makers and solution providers [1, 7]. Effective teaching and learning is now considered as one which involves active immersion and participation by students and not just a scenario in which students are mere passive listeners [8, 9]. This development in the tertiary educational system not only lends credence to deeper concentration on students but also to serious considerations being given to their perceptions and feed-backs on teachers’ qualities and on the teaching methods used in teaching and impacting knowledge in them.

There is evidence that teachers exhibit certain qualities and characteristics in the art of teaching as documented in publications as far back as the 1960s [10]. However, students, being the recipients of knowledge during the teaching process also have perceptions of what they consider as effective teaching methods and what they consider as effective qualities which they desire to see in a teacher [1]. The assumption that effective teaching and effective learning cannot be accurately measured or defined and the divergent views held on what qualifies as effective learning among scholars have led researchers to conduct studies on identifying elements of teaching and characteristics of teachers which may be useful in making students’ learning possible, enthusiastic and deep. The feedback obtained from students will not merely serve to obtain their perspectives; it will also be taken into cognizance in proposing reforms and changes that need to be made to improve the teaching and learning processes in tertiary educational systems. We investigated students’ perceptions of teachers among a cohort of newly admitted students at the University of Medical Sciences in Ondo State, South-west Nigeria. The objective of this study was to document the views expressed by students regarding what they expect of their teachers in curricular delivery and social interactions. We believe the methodology and results will be useful for tertiary institutions that often struggle with identifying ways to obtain feedback from students in the development and review of teaching curricular.

## Methods

This was a descriptive quantitative study consisting of community-interactive session with students at the 100 and 200 levels of the University of Medical Sciences (UNIMED) in Ondo city, Ondo State of Nigeria. The University of Medical Sciences is the first specialized university in health sciences disciplines in Nigeria. It was established by the Ondo State Government (one of the 36 States in the Federal Republic of Nigeria) in 2014 and approved by the Nigerian regulatory agency for tertiary institutions, the National Universities Commission (NUC) in April 2015. The university offers courses in the biological and physical sciences, basic medical sciences, clinical medicine, pathological sciences, dentistry, physiotherapy, medical laboratory sciences and nursing. The first set of students came into residence in January 9, 2016. The mission of UNIMED is: “to provide exceptional quality and comprehensive health care and integrated education and research in all health-related sciences, to expand access to compassionate and high quality health care for under-served populations, and to lead the scientific pathway for reducing the burden of disease in our areas of operation” [11]. The vision of the university is “to be a thriving medical and health sciences university, locally, nationally and internationally recognized for excellence and innovation in health education and research, and for its ability to translate research findings for the improvement of health in communities with high burden of disease” [11].

In efforts to achieve its mission and vision statements, the university management decided from the onset to develop teaching curricular that meet student’s interest and demand for training. These interactive session and research were therefore part of the strategies adopted by the new university for developing a functional and students-friendly curricular. We took opportunity of the annual orientation week organized for new and returning students in the university in January 2017 to conduct the study. Three hundred (300) students participated in the interactive session that took place in the main auditorium of the university. The students were addressed by a senior staff member of the Academic Planning Unit of the University, who was not an active teaching faculty in the university. The purpose of the study was fully explained to the students, and only those who agreed to participate in the fully explained study were allowed to continue in the interactive session. They were specifically informed that the study was designed to obtain information for improving the teaching quality in the university. The students were requested to answer the questions truthfully and independently, and they were assured of confidentiality of information obtained from them. The students were asked not to include their names or any identification numbers in the questionnaires. Thereafter, the students individually completed a semi-structured questionnaire that elicited information on their views about the qualities and distinctive traits they expected from their lecturers. The responses were aggregated under the following sub-heads: expertise, approachability, good communication skills, teaching skills, friendliness and congeniality, enthusiasm, good sense of humor, good personality, vision of high expectations, good class management with effective discipline skills, mentorship, equity, punctuality, good time management and non-abusive and cursing. Of these attributes, the students were asked to pick those that mattered to them the most. Additional provisions were made for them to explain the reasons for their choices and also to add more attributes that were not captured in the list provided.

**Ethical consideration:** ethical approval was obtained from the Ethical Committee of University of Medical Sciences. Informed consent was obtained from each participant. All questionnaires were coded (without names) and confidentiality of responses was ensured throughout the study.

**Data analysis:** data analysis was done with SPSS version 21. The frequency and percentage distribution of the aggregated responses were calculated and ranked to determine the attributes that were most important to the students. The open-ended questions were analyzed qualitatively for content and themes, and reported under various thematic categories.

## Results

**Figure 1:**
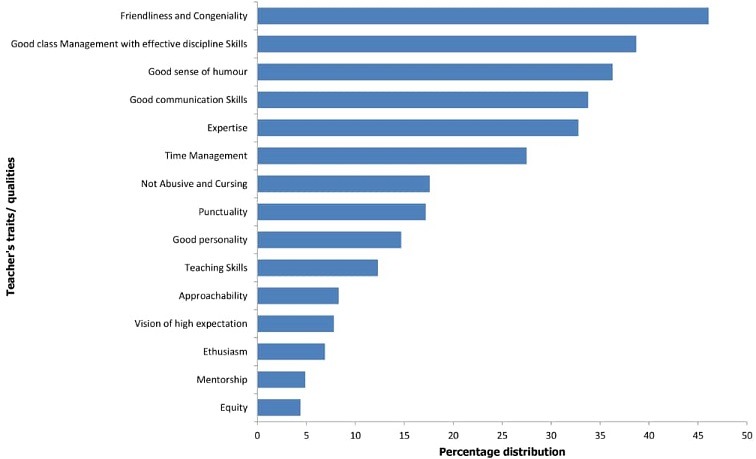
Percentage distribution of the traits/qualities in descending order of magnitude

Of the 300 students that participated in the community-interactive session, 204 (68%) completed the questionnaires and made submissions that were analyzed. The quantitative analysis of the structured questionnaire is presented in Table 1. The results showed that the students rated friendliness and congeniality, good classroom management with effective disciplinary skills, good sense of humor, good communication skills, and expertise as the five most desirable traits of a good lecturer. These traits scored 46.1%, 38.7%, 36.3%, 33.8% and 32.8% respectively. By contrast, the five traits which rated lowest in the assessment of the students included equity (4.4%), mentorship (4.9%), enthusiasm (6.9%), pushing students to succeed (7.8%) and approachability (8.3%) respectively (Figure 1).

**Table 1:** Frequency and percentage distribution of student’s response

Expected Qualities	Frequency	%	Total
**Friendliness and congeniality** *(kind and have strong rapport with students, allow students to share their problems without being afraid or hesitant)*	94	46.1	204
**Good class management with effective disciplinary skills** *(ensure good student behaviour, effective study and work habits and overall sense of respect in the class)*	79	38.7	204
**Good sense of humor** *(the quality of being funny)*	74	36.3	204
**Good communication skills** *(use of right word to gain access to contents of student’s minds, tailor messages to best suit students’ language abilities and preferences)*	69	33.8	204
**Expertise** *(knowledge of subject matter, knowledge of curriculum and standards, intellectual curiosity, clear objectives for lessons, awareness of changes in subject area, confidence)*	67	32.8	204
**Good time management** *(doesn’t exceed lecture schedule or devote lecture schedule to irrelevant activities)*	56	27.5	204
**Not abusive or cursing**	36	17.6	204
**Punctuality** *(timely attendance at lecture)*	35	17.2	204
**Good personality** *(good, decent, likeable, dress sensibly well, being a little gentle and kind)*	30	14.7	204
**Teaching skills** *(selection of appropriate course contents, give lessons logical structure)*	25	12.3	204
**Approachability** *(a good listener, patience, Maturity)*	17	8.3	204
**Pushing students to succeed** *(encourages students to work at their best level)*	16	7.8	204
**Enthusiasm** *(transmit excitement and interest in the subject)*	14	6.9	204
**Mentorship** *(the desire to influence students positively)*	10	4.9	204
**Equity** *(not partial in dealing with male and female students)*	9	4.4	204

## Discussion

The study was designed to explore the students’ expectations of their teachers in a specialized University of Medical Sciences in Nigeria. The results showed that students were more likely to rate non-academic attributes of teachers such as their being friendly as compared to specific academic attributes. It was of interest that in the ranking of the results, the students ranked attributes such as “friendliness and congeniality” and a “sense of humor”, ahead of qualities such as “expertise” and “teaching skills”. Even “mentorship”, which is a critical skill, required of teachers in tertiary educational institutions was lowly rated by the students. Similar results have been reported in a study on students’ assessment of teachers in other parts of sub-Saharan Africa [12]. A study reported by Adomi [12] from Southern Nigeria showed that students highly rated good knowledge of the course, dressing well, punctuality, friendliness, and love for students in their perceptions of teacher effectiveness, while practical knowledge and pedagogical skills were ranked low. Also, the results of this study agrees with that of Voss *et al.* [13] who reported friendliness and knowledge as some of the most desired quality in a teacher by a set of German students. However, unlike our study where enthusiasm and approachability were rated low as desired teachers’ qualities, these qualities were rated high in the study by Voss *et al.* [13]. Teaching skills was also one of the most desired teachers’ qualities by the students in our study which is similar to reports by Sander *et al.* [14].

Although students in developed countries frequently participate in the evaluation of their teachers, very few studies reported students’ perceptions regarding their expectations of teachers. It would appear that these expectations are taken for granted in developed countries. Murray *et al.* [15] in a detailed review identified some of the best qualities of teachers in tertiary institutions as “content competence, pedagogical competence, the ability to deal with sensitive topics in an open, honest and respectful manner, the ability to contribute to intellectual development of the student, the ability to treat students’ grades and private communications/conversations with strict confidentiality, and the assessment of students that is valid, open, fair and congruent with the course and respect of the institution”. It was of interest that none of these ethical teaching principles featured in this study as students’ expectations of their teachers. The results of this study have implications for the design of training programs for teachers in tertiary educational systems in Nigeria. It also has consequences for the design of policies for the evaluation of teachers in tertiary institutions. It suggests that training curricular for teachers must not only include pedagogical skills, it should also be expanded to include teacher-student interactions on social and personal issues.

Similarly, protocols designed for students to evaluate their teachers in contexts where social issues predominate should be structured rather than being open-ended. By being structured, it would ensure that the real components of pedagogical and academic skills are not left out in the assessment of teachers by students, while also incorporating the social components that students are most concerned about. The major strength of this study was the design which enabled the students to provide answers in a value-free manner and in their own understanding of the subject matter. None of the teaching faculties of the university participated in the community interactions, allowing open and frank exposure of the issues and the completion of the study protocol in a non-intimidating and open way. By contrast, the main limitation of the study is its conduct in one institution only, especially in a specialized institution that is restricted to teaching health sciences courses. It is therefore possible that the results cannot be generalized to the wider context of tertiary education in Nigeria and other African countries that have large population of students from diverse disciplines. Also, the study was conducted with new students at junior classes of the University, which suggests that the students may not have had the time and experience to discuss expectations of their teachers in an evidence-based manner. Despite the limitations, we believe that the results are thought-provoking and would be useful for improving curricular delivery and the quality of teaching in the tertiary educational institutions in resource-poor countries.

## Conclusion

We conclude that students at the University of Medical Sciences look out for personal social relationships with their teachers during curricular delivery. We recommend that approaches to address these concerns should be integrated into the design of training programs for teachers and in protocols for students’ evaluation of teachers in this university and others in similar circumstances.

### What is known about this topic

- The assessment of teachers by students has featured as an indicator for measuring the quality of curricular delivery in tertiary institutions;
- The modern trend in tertiary education is to shift from the traditional didactic, teacher-focused teaching to student-centered methodologies that encourage the active engagement of students in the learning process.

### What this study adds

- Students look out for personal and social relationships with their teachers during curricular delivery;
- Students are more likely to rate non-academic attributes of teachers as compared to specific academic attributes;
- Training curricular for teachers must not only include pedagogical skills, but also be expanded to include teacher-student interactions on social and personal issues.

## Competing interests

The authors declare no competing interests.
